# Objectively measured sedentary time, physical activity and kidney function in people with recently diagnosed Type 2 diabetes: a prospective cohort analysis

**DOI:** 10.1111/dme.12886

**Published:** 2015-09-08

**Authors:** V. YW. Guo, S. Brage, U. Ekelund, S. J. Griffin, R. K. Simmons, the ADDITION‐Plus study team, R. Amin, G. Baker, M. Betts, A. Dickinson, J. B. Echouffo Tcheugui, F. Finucane, S. Mayle, J. Mitchell, P. Roberts, L. Sargeant, M. Sims, K. Westgate, J. Argles, R. Bale, R. Barling, S. Boase, J. Brimicombe, R. Butler, T. Fanshawe, P. Gash, J. Grant, W. Hardeman, I. Hobbis, A. L. Kinmonth, T. McGonigle, N. Popplewell, A. T. Prevost, J. Smith, M. Smith, S. Sutton, F. Whittle, K. Williams

**Affiliations:** ^1^ MRC Epidemiology UnitUniversity of Cambridge School of Clinical MedicineCambridgeUK; ^2^ Division of BiostatisticsSchool of Public HealthFaculty of MedicineThe Chinese University of Hong KongShatinNew TerritoriesHong Kong; ^3^ Department of Sports MedicineNorwegian School of Sport SciencesOsloNorway; ^4^ The Primary Care UnitInstitute of Public HealthUniversity of CambridgeCambridgeUK

## Abstract

**Aim:**

To assess the prospective association between objectively measured physical activity and kidney function over 4 years in people with Type 2 diabetes.

**Methods:**

Individuals (120 women and 206 men) participating in the ADDITION‐Plus trial underwent assessment of sedentary time (SED‐time), time spent in moderate‐to‐vigorous‐intensity physical activity (MVPA) and total physical activity energy expenditure (PAEE) using a combined heart rate and movement sensor, and kidney function [estimated glomerular filtration rate (eGFR), serum creatinine and urine albumin‐to‐creatinine ratio (ACR)] at baseline and after 4 years of follow‐up. Multivariate regression was used to quantify the association between change in SED‐time, MVPA and PAEE and kidney measures at four‐year follow‐up, adjusting for change in current smoking status, waist circumference, HbA_1c_, systolic blood pressure, triglycerides and medication usage.

**Results:**

Over 4 years, there was a decline in eGFR values from 87.3 to 81.7 ml/min/1.73m^2^ (*P* < 0.001); the prevalence of reduced eGFR (eGFR < 60 ml/min/1.73m^2^) increased from 6.1 to 13.2% (*P* < 0.001). There were small increases in serum creatinine (median: 81–84 μmol/l, *P* < 0.001) and urine ACR (median: 0.9–1.0 mg/mmol, *P* = 0.005). Increases in SED‐time were associated with increases in serum creatinine after adjustment for MVPA and cardiovascular risk factors (β = 0.013, 95% CI: 0.001, 0.03). Conversely, increases in PAEE were associated with reductions in serum creatinine (β = –0.001, 95% CI: –0.003, –0.0001).

**Conclusion:**

Reducing time spent sedentary and increasing overall physical activity may offer intervention opportunities to improve kidney function among individuals with diabetes. (Trial Registry no. ISRCTN 99175498)

## What's new?

- Little is known about the prospective association between objectively measured physical activity and kidney function among individuals with Type 2 diabetes.
- Over 4 years of follow‐up, reductions in sedentary time and increases in total physical activity energy expenditure were associated with reductions in serum creatinine.
- Reducing time spent sedentary and increasing overall physical activity may offer intervention opportunities to improve kidney function among individuals with diabetes.

**What's new?**

- Little is known about the prospective association between objectively measured physical activity and kidney function among individuals with Type 2 diabetes.
- Over 4 years of follow‐up, reductions in sedentary time and increases in total physical activity energy expenditure were associated with reductions in serum creatinine.
- Reducing time spent sedentary and increasing overall physical activity may offer intervention opportunities to improve kidney function among individuals with diabetes.

## Introduction

Diabetes is the leading cause of kidney disease 1, which is associated with increased risk of cardiovascular events and mortality 2. Low levels of total physical activity energy expenditure (PAEE) and long time spent sedentary (SED‐time) are associated with higher metabolic risk in patients with diabetes 3. Being physically inactive is also associated with kidney dysfunction, in both the general population 4, 5, 6, 7, 8 and among individuals with diabetes 9, 10. However, most studies to date are cross‐sectional and rely on self‐reported measures of physical activity, which are prone to error and bias. To the best of our knowledge, there are no studies that examine the prospective association between objectively assessed physical activity and kidney function among individuals with diabetes.

We aimed to assess the association between changes in objectively measured moderate‐to‐vigorous‐intensity physical activity (MVPA), SED‐time and PAEE over 4 years with kidney function in a cohort of individuals with recently diagnosed Type 2 diabetes 11. These individuals typically have low levels of physical activity, and spend limited time in MVPA and a large amount of time being sedentary 3, 12. Improved understanding of this relationship will inform the development of interventions to reduce the risk of kidney dysfunction in this high‐risk group.

## Methods

ADDITION‐Plus is an explanatory randomized controlled trial, nested within the intensive treatment arm of ADDITION‐Cambridge study 11, 13. Eligible participants were those aged 40–69 years who were diagnosed with Type 2 diabetes following screening in the ADDITION‐Cambridge study or clinically diagnosed during the previous 3 years. A total of 478 eligible participants were randomly assigned to either intensive treatment alone (*n* = 239) or intensive treatment plus a facilitator‐led behaviour change intervention delivered at the patient's practice (*n* = 239). Details of the intervention have been described previously 11. Because no differences were observed in the main outcomes after 1 year, the two trial arms were pooled to conduct a cohort analysis 3, 14. All participants provided written informed consent. The study was approved by the Eastern Multi‐Centre Research Ethics Committee (reference number: 02/5/54). The trial was registered as ISRCTN99175498.

### Assessment of physical activity and kidney function

Assessment of ADDITION‐Plus participants included identical physiological and anthropometric measures by trained staff following standard operating procedures at baseline, 1 and 5 years. We used data from 1 and 5 years (hereafter referred to as baseline and follow‐up) as objectively measured physical activity was only assessed at 1 and 5 years.

We measured physical activity using a combined heart rate (HR) and movement monitor (Actiheart, CamNtech, Cambridge, UK), worn continuously for at least 4 days, collecting data in 30s resolution 15. A graded treadmill walk test was used to calibrate HR against known workload individually 16. HR data collected during the free‐living period were processed using a two‐stage method of noise classification followed by Gaussian robust regression 17. Average activity intensity was estimated using branched equation modelling 18. Resulting time‐series data were summarized into MVPA (h/day), SED‐time (h/day) and PAEE (kJ/kg/day), whilst minimizing diurnal information bias caused by non‐wear periods (segments of non‐physiological data). MVPA was defined as intensity ≥ 3.0 metabolic equivalent task (MET) and SED‐time as intensity < 1.5 MET excluding self‐reported sleep duration using a standard definition of 1 MET = 3.5 ml O_2_/min/kg^ ^× 20.35 J/ml O_2_. For participants without the individual calibration test, data collected during the free‐living period were processed with a group calibration equation adjusted for age, sex, β–blocker and sleeping HR in order to translate HR into activity intensity derived on the basis of all individuals with a treadmill test. Self‐reported habitual bedtime and wake time were evaluated on weekdays and weekends separately using the EPIC‐Norfolk Physical Activity Questionnaire (EPAQ2) 19. Sleep duration was calculated as (5/7 × weekday sleep duration) + (2/7 × weekend sleep duration).

Urine albumin‐to‐creatinine ratio (ACR) was measured with a spot urine sample. Micro‐albuminuria was defined as urine 2.5 ≤ ACR < 25 mg/mmol in men or 3.5 ≤ ACR < 25 mg/mmol in women. Macro‐albuminuria was defined as urine ACR ≥ 25 mg/mmol. Serum creatinine was assessed using kinetic colorimetric methods. Estimated GFR (eGFR) was calculated on the basis of age, gender and serum creatinine using the Modification of Diet in Renal Disease formula 20. Decreased eGFR was defined as eGFR < 60 ml/min/1.73m^2^. Chronic kidney disease (CKD) was defined as decreased eGFR and/or albuminuria (the presence of either micro‐albuminuria or macro‐albuminuria).

### Covariate assessment

Standardized self‐report questionnaires were used to collect socio‐economic characteristics, smoking and alcohol status. Dietary intake was evaluated using a validated food frequency questionnaire 21. The data were subsequently converted into total energy intake (kJ/day and kcal/day) and fat intake (g/day).

Waist circumference was assessed halfway between the lowest point of the costal margin and the anterior superior iliac crest while standing. Blood pressure (BP) was assessed after 10 min of rest using an automatic sphygmomanometer (Omron M4; Milton Keynes, UK). Hypertension was defined as systolic BP/diastolic BP ≥ 140/90 mmHg, with/without anti‐hypertensive treatment. HbA_1c_ was measured in venous samples by ion‐exchange high‐performance liquid chromatography (Tosoh Bioscience, Redditch, UK). Serum total cholesterol, triglycerides and HDL‐cholesterol were measured using enzymatic techniques (Dimension Analyzer; Dade Behring, Newark, NJ, USA). LDL‐cholesterol was calculated for each individual using the Friedewald equation 22.

We excluded individuals who did not have complete data for physical activity parameters at baseline and follow‐up (*n* = 113), for clinical and socio‐economic information (*n* = 21), for kidney function at baseline and follow‐up (*n* = 14) and individuals who wore the activity monitor for < 48 h (*n* = 4). The present analyses therefore included 326 participants.

### Statistical analysis

To compare the differences between baseline and follow‐up, paired *t*‐tests or Wilcoxon signed rank tests were used for continuous data, and McNemar's test was used for categorical variables. We also compared baseline socio‐economic and clinical characteristics between included individuals (*n* = 326) and those excluded (*n* = 152).

Multivariate linear regression analysis was used to examine the association between changes (∆) in MVPA, SED‐time and PAEE (defined as follow‐up values minus baseline values) with eGFR, serum creatinine and urine ACR at follow‐up. Serum creatinine and urine ACR were log‐transformed in order to meet the assumption of linear regression. Model 1 was adjusted for baseline age, sex, socio‐economic status, baseline MVPA (when the exposure was ∆MVPA), baseline SED‐time (when the exposure was ∆SED‐time), baseline PAEE (when the exposure was ∆PAEE), baseline eGFR (when the outcome was eGFR at follow‐up), baseline serum creatinine (when the outcome was serum creatinine at follow‐up), and baseline urine ACR (when the outcome was urine ACR at follow‐up). Model 2 was further adjusted for baseline and ∆SED‐time (when ∆MVPA was the exposure) or baseline and ∆MVPA (when ∆SED‐time was the exposure). Model 3 was further adjusted for changes in cardiovascular risk factors, namely current smoking status, waist circumference, systolic BP, HbA_1c_, serum triglycerides and medication usage, including glucose‐lowering, lipid‐lowering, anti‐hypertensive and aspirin medication. Linearity, normality, homoscedasticity and absence of multicollinearity were checked for all models. In a secondary analysis we used multivariable logistic regression to explore the associations between changes in SED‐time, MVPA and PAEE between baseline and follow‐up with the dichotomous outcome of CKD at follow‐up adjusting for the same variables above and the additional adjustment of baseline CKD status. Statistical analyses were performed using Stata/SE 12.0 (Stata‐Corp, College Station, TX, USA). Statistical significance was set at *P* < 0.05.

## Results

Among the participants included in this analysis (n = 326), the mean (standard deviation [SD]) age was 61.2 (7.1) years. Median duration of diabetes was 1.4 years (interquartile range [IQR]: 1.1−2.9). The majority were male (63%) and from a managerial/professional socio‐economic class (43%). Compared to those included, excluded participants (n = 152) reported shorter sleep duration and had longer sedentary and shorter MVPA time at baseline. There was no difference in kidney function or other baseline characteristics between included and excluded participants (data not shown).

After 4 years of follow‐up, participants reported lower total energy intake and longer sleep duration at follow‐up compared with baseline (Table 1). There was a small increase in BMI for men. Mean HbA_1c_ levels increased from 49 to 53 mmol/mol (6.6 to 7.0%), while diastolic BP, total cholesterol and LDL‐cholesterol levels declined. The prevalence of hypertension increased from 77.3 to 84.7%. There was a small but significant increase in HDL‐cholesterol in the whole cohort. The proportion of participants prescribed glucose‐lowering, lipid‐lowering and anti‐hypertensive medication increased significantly during the follow‐up period.

**Table 1:** Characteristics of the ADDITION‐Plus cohort at baseline and follow‐up (*n* = 326)

Characteristic	Baseline	Follow‐up	*P*
**Demographic and lifestyle factors**			
Age (years)	61.2 (7.1)	−	−
Male sex (%)	63.2	−	−
Occupational socio‐economic class (%)		−	−
Managerial and professional	42.9		
Intermediate	24.5		
Routine and manual	32.5		
Alcohol consumption (g/day)^a^	3.8 (0.1, 10)	2.0 (0, 10.0)	0.213
Current smoker (%)	12.0	11.7	1.000
Fat intake (g/day)	59.5 (20.8)	60.5 (25.9)	0.429
Total energy intake (kJ/day)^a^	7162.3 (6044.4, 8451.1)	6644.0 (5686.4, 8094.4)	0.009
Total energy intake (kcal/day)^a^	1698.9 (1431.5, 2001.7)	1579.5 (1347.0, 1917.8)	0.010
Average sleep duration (h/day)^b^	8.2 (1.1)	8.4 (1.0)	< 0.001
**Clinical/biochemical measures**			
BMI (kg/m^2^)			
Female	32.2 (5.5)	32.3 (5.7)	0.510
Male	31.3 (4.9)	31.6 (5.4)	0.015
Waist circumference (cm)			
Female	103.7 (12.6)	102.5 (12.8)	0.063
Male	110.5 (12.6)	110.5 (13.2)	0.961
Obesity (%)	57.7	58.3	0.883
HbA_1c_ [mmol/mol, (%)]	49 (10) [6.6 (0.9)]	53 (10) [7.0 (0.9)]	< 0.001
Hypertension (%)	77.3	84.7	< 0.001
Systolic BP (mmHg)	130.0 (17.7)	131.8 (16.6)	0.054
Diastolic BP (mmHg)	76.0 (9.1)	73.3 (9.2)	< 0.001
Total cholesterol (mmol/l)	4.3 (0.9)	4.1 (0.9)	0.013
Triglycerides (mmol/l)^a^	1.6 (1.1, 2.2)	1.6 (1.2, 2.3)	0.359
HDL‐cholesterol (mmol/l)	1.2 (0.3)	1.3 (0.3)	< 0.001
LDL‐cholesterol (mmol/l)	2.3 (0.7)	2.1 (0.7)	< 0.001
**Kidney measures**			
eGFR (ml/min/1.73m^2^)	87.3 (29.2)	81.3 (23.3)	< 0.001
Decreased eGFR (%)	6.1	13.2	< 0.001
Urine ACR (mg/mmol)^a^	0.9 (0.5, 1.8)	1.0 (0.6, 2.1)	0.005
Micro‐albuminuria (%)	13.8	16.9	0.184
Macro‐albuminuria (%)	1.5	2.5	0.453
Serum creatinine (μmol/l)^a^	81 (69, 91)	84 (72, 93)	< 0.001
CKD (%)	19.0	27.3	0.001
**Prescribed medication**			
Glucose‐lowering medication (%)	48.9	71.2	< 0.001
Lipid‐lowering medication (%)	77.6	85.3	0.003
Anti‐hypertensive medication (%)	73.5	78.8	< 0.001
Aspirin (%)	56.4	56.4	1.000
**Objectively measured health behaviours**			
PAEE (kJ/kg/day)	33.7 (17.0)	28.7 (15.3)	< 0.001
Duration SED‐time (h/day)	10.5 (2.5)	11.1 (2.1)	< 0.001
Duration MVPA (h/day)^a^			
Female	0.23 (0.09, 0.61)	0.18 (0.04, 0.50)	0.191
Male	0.59 (0.27, 1.23)	0.49 (0.17, 1.03)	0.005

All data are means (sd) or percentage unless otherwise indicated.

^a^Median (IQR).

^b^Sleep duration was calculated using the self‐reported EPIC‐Norfolk Physical Activity Questionnaire (EPAQ2).

Obesity was defined as BMI > 30 kg/m^2^. Hypertension was defined as systolic BP/diastolic BP ≥ 140/90 mmHg, with or without anti‐hypertension treatment.

There was a decline in eGFR values from 87.3 to 81.7 ml/min/1.73m^2^ (*P* < 0.001); the prevalence of decreased eGFR increased from 6.1 to 13.2% (*P* < 0.001). There were small increases in urine ACR from a median of 0.9 to 1.0 mg/mmol (*P* = 0.005) and serum creatinine from a median of 81 to 84 μmol/l (*P* < 0.001). There was no significant increase in the proportion of participants with micro‐ or macro‐albuminuria. The prevalence of CKD was 19.0% at baseline and 27.3% at follow‐up (*P* = 0.001).

In terms of objectively measured health behaviours, PAEE levels declined from 33.7 to 28.7 kJ/kg/day (*P* < 0.001), whereas SED‐time increased over the 4–year follow‐up (10.5 vs. 11.1, *P* < 0.001). Time spent in MVPA was similar at baseline and follow‐up for women, whereas men showed a significant reduction in MVPA (median: 0.59 to 0.49 h/day, *P* = 0.005).

There was a significant inverse association between change in SED‐time and eGFR at follow‐up in Model 1 (Table 2). However, after adjustment for MVPA and cardiovascular risk factors (Models 2 and 3), these associations were attenuated. By contrast, change in PAEE was positively associated with eGFR at follow‐up in Model 1. This association was no longer significant after further adjustment of cardiovascular risk factors (Model 3). There was no significant association between changes in MVPA and eGFR at follow‐up in any of the models. There was a positive association between change in SED‐time and serum creatinine at follow‐up in all adjusted models, e.g. on average, participants who increased their SED‐time between baseline and follow‐up had increased levels of creatinine at follow‐up. Conversely, increased PAEE was inversely associated with serum creatinine at follow‐up in all adjusted models. There was no association between changes in MVPA and creatinine. There were no significant associations between change in SED‐time, MVPA or PAEE and urine ACR at follow‐up.

**Table 2:** Association between change in MVPA, SED‐time and PAEE from baseline to four‐year follow‐up and kidney function at 4 years in the ADDITION‐Plus trial cohort

Exposure		eGFR at follow‐up		log (Serum creatinine) at follow‐up		log (Urine ACR) at follow‐up	
		β (95% CI)	*P*	β (95% CI)	*P*	β (95% CI)	*P*
Model 1	ΔSED‐time	–0.984 (–1.893, –0.076)	0.034	0.012 (0.003, 0.022)	0.012	–0.030 (–0.090, 0.029)	0.314
	ΔMVPA	2.204 (–0.419, 4.827)	0.099	–0.020 (–0.048, 0.008)	0.166	–0.024 (–0.195, 0.146)	0.779
	ΔPAEE	0.153 (0.018, 0.288)	0.026	–0.002 (–0.003, –0.0002)	0.025	0.002 (–0.007, 0.008)	0.706
Model 2	ΔSED‐time	–0.773 (–1.861, 0.316)	0.163	0.012 (0.0003, 0.024)	0.045	–0.049 (–0.120, 0.022)	0.179
	ΔMVPA	1.012 (–2.121, 4.145)	0.525	–0.001 (–0.035, 0.033)	0.954	–0.101 (–0.305, 0.102)	0.327
Model 3	ΔSED‐time	–0.791 (–1.894, 0.313)	0.159	0.013 (0.001, 0.025)	0.030	–0.048 (–0.120, 0.023)	0.187
	ΔMVPA	0.475 (–2.694, 3.643)	0.768	0.008 (–0.026, 0.043)	0.652	–0.119 (–0.304, 0.066)	0.171
	ΔPAEE	0.128 (–0.009, 0.266)	0.067	–0.001 (–0.003, –0.0001)	0.048	–0.001 (–0.009, 0.009)	0.977

Model 1 was adjusted for age, sex, socio‐economic status, baseline value of the relevant outcome, and baseline value of the relevant exposure.

Model 2 was further adjusted for baseline SED‐time and ΔSED‐time (when ΔMVPA was the exposure) or baseline MVPA and ΔMVPA (when ΔSED‐time was the exposure).

Model 3 was further adjusted for Δcurrent smoking status, Δwaist, ΔHbA_1c_, Δsystolic BP, Δtriglycerides, Δglucose‐lowering drugs, Δlipid‐lowering drugs, Δanti‐hypertensive drugs and Δaspirin from baseline to 4 years.

When examining kidney dysfunction as a binary outcome at 4–year follow‐up, there was no association between change in MVPA or change in SED‐time with CKD in fully adjusted models (Table 3). An increase in PAEE was significantly associated with a reduction in the relative risk of developing CKD in all models (Model 3: relative risk: 0.964, 95% CI: 0.936–0.993, *P* = 0.014, for every 1 kJ/kg/day difference in PAEE change).

**Table 3:** Multivariable logistic regression analysis of the association between change in MVPA, SED‐time and PAEE from baseline to 4–year follow‐up and CKD at four years in the ADDITION‐Plus trial cohort

		Relative risk	95% CI	*P*
Model 1	ΔSED‐time	1.149	0.968–1.364	0.113
	ΔMVPA	0.544	0.294–1.007	0.053
	ΔPAEE	0.968	0.941–0.995	0.023
Model 2	ΔSED‐time	1.095	0.887–1.352	0.397
	ΔMVPA	0.689	0.333–1.428	0.316
Model 3	ΔSED‐time	1.088	0.867–1.365	0.466
	ΔMVPA	0.591	0.272–1.282	0.183
	ΔPAEE	0.964	0.936–0.993	0.014

Model 1 was adjusted for age, sex, socio‐economic status, baseline CKD status and baseline value of the relevant exposure.

Model 2 was further adjusted for baseline SED‐time and ΔSED‐time (when ΔMVPA was the exposure) or baseline MVPA and ΔMVPA (when ΔSED‐time was the exposure).

Model 3 was further adjusted for Δcurrent smoking status, Δwaist, ΔHbA_1c_, Δsystolic BP, Δtriglycerides, Δglucose‐lowering drugs, Δlipid‐lowering drugs, Δanti‐hypertensive drugs and Δaspirin from baseline to 4 years.

## Discussion

Among a cohort of individuals with recently diagnosed diabetes, increases in sedentary time over 4 years were associated with increases in serum creatinine after adjustment for time spent in moderate‐to‐vigorous‐intensity physical activity and cardiovascular risk factors. Conversely, increases in total physical activity energy expenditure were associated with reductions in serum creatinine and with a lower risk of developing CKD.

Previous research on the association between physical activity and kidney function comes primarily from the general population. The National Health and Nutrition Examination Survey (NHANES) II showed that self‐reported inactive individuals had a higher risk of CKD‐related deaths and end‐stage renal disease compared with active individuals 6. In a Norwegian population without signs of CKD at baseline, high levels of self‐reported physical activity at baseline were associated with increases in eGFR only in women after 7 years of follow‐up 4. However, a report from the Australian Diabetes, Obesity and Lifestyle Study (AusDiab) of 5853 participants who were free of CKD at baseline found no significant association between self‐reported physical activity and CKD incidence after 5 years of follow‐up 8. We were also unable to demonstrate any significant association between change in objectively measured time spent in moderate‐to‐vigorous‐intensity physical activity or total energy expenditure and eGFR at 4–year follow‐up. In terms of sedentary time and kidney function, the AusDiab study showed that television‐viewing time was a risk factor for low eGFR in men over 5 years of follow‐up 5, although the association between physical activity and eGFR varied across previous studies. Overall, these studies suggest a protective role of physical activity on kidney function.

One cross‐sectional study in 3587 women without diabetes found an inverse association between self‐reported time spent walking and strenuous activity with albuminuria 7. However, the AusDiab study of 5978 participants at risk of albuminuria showed that self‐reported physical activity was not associated with long‐term albuminuria 8. In patients with Type 2 diabetes, one small intervention study with 30 male participants, who underwent regular aerobic exercise for 6 months, showed reduced urine ACR after 6 months follow‐up 10. In this analysis, there was no evidence of an association between any physical activity measures and ACR.

Only one small study of 17 patients with CKD has focused on the effect of exercise on serum creatinine level 23. Participants undergoing water‐based exercise showed reduced serum creatinine during a period of 12 weeks. In our study, sedentary time and total energy expenditure were associated with serum creatinine levels.

Our results are not fully consistent with previous studies. One possible explanation might be the different method for assessing physical activity. Most of the reports used questionnaires to collect the frequency, duration and intensity of physical activity 4, 5, 6, 7, 8, 9, 10. Information on physical activity derived from self‐reported questionnaires is subject to error and social desirability bias or cognitive limitations due to recall or comprehension 24. A second possible reason might be the different study populations. Most studies focused on the general population 4, 5, 6, 7, 8 and only a few studies were conducted among individuals with diabetes 9, 10. Our cohort focused on patients in the first 5 years after diagnosis of diabetes. If we had followed our cohort for longer, we may have discovered stronger relationships between physical activity and kidney function. Finally, people with recently diagnosed diabetes spend significant amounts of time in sedentary behaviours with limited time spent in moderate‐to‐vigorous‐intensity physical activity 3. Thus, we observed the strongest associations between sedentary time and total energy expenditure with kidney function. The lack of association between time spent in moderate‐to‐vigorous‐intensity physical activity and outcome may, therefore, be explained by low and homogenous levels of physical activity in the study population.

The strengths of our study include the prospective design, and validated methods to assess exposure, outcomes and covariates. We measured physical activity objectively using a combined HR and movement sensor, which allowed us to discriminate between time spent in different intensities of physical activity. This is crucial to accurately estimate sedentary time in recently diagnosed Type 2 diabetes patients, who spend a considerable amount of time in sedentary behaviours. Furthermore, the device used in this study has been demonstrated a reliable and valid tool 15, and the use of branched equation modelling improves the precision of estimates of intensity as well as total energy expenditure 18, 25. We also focused on individuals with recently diagnosed Type 2 diabetes. Because diabetes duration is a key contributor to kidney dysfunction 26, our study may provide intervention points for preventing or slowing the progression of kidney dysfunction.

Several limitations should also be addressed. First, urine ACR was measured only once at each time point using random spot urine sample. Potential bias may be present in patients with high protein excretion when estimating urine ACR 27, which might attenuate the associations between physical activity and urine ACR. Second, we did not consider time spent napping during the day, which might lead to misclassification of sleep time as sedentary time and vice versa. Also, participants may misreport sleep duration. A more precise measure of sleep duration, such as polysomnography recording biophysiological changes during sleep, might reduce this bias, but is unlikely to be carried out among free‐living participants in large‐scale epidemiological cohorts. Third, although we adjusted for conventional risk factors for kidney dysfunction, we cannot rule out other possible confounders or unmeasured factors such as changes in lean mass and dietary intake of creatinine. Conversely, the adjustment of cardiovascular risk factors may have attenuated the association between physical activity measures and kidney function. Fourth, this was a post‐hoc question and we carried out a number of statistical tests on multiple exposure‐outcome relationships, which may have led to spurious positive results. Finally, our study was conducted in a relatively homogeneous sample with the same ethnic background; our results may therefore not be generalizable to other populations.

Being physically active reduces cardiovascular disease and total mortality in patients with Type 2 diabetes 28, and early intervention targeted at increasing physical activity may be an effective strategy to reduce the risk of later macrovascular complications. We previously showed that objectively measured sedentary time was associated with metabolic risk factors independent of time spent in moderate‐to‐vigorous‐intensity physical activity 3. Focusing on reducing sedentary behaviour and/or increasing total physical activity energy expenditure, rather than solely targeting increasing the time spent on physical activity, might be a more effective paradigm to improve health outcomes. This is particularly important because patients with Type 2 diabetes spend a long time sitting and less time being physically active. Our findings suggest that increasing total physical activity energy expenditure, regardless of activity intensity, has beneficial effect on kidney function. Our data also suggest that apart from the World Health Organization guidelines of spending at least 150 min on moderate‐to‐vigorous‐intensity physical activity per week, interventions that increase total physical activity energy expenditure by reducing sedentary time may also benefit patients. For example, an increase of 6 kJ/kg/day energy expenditure, which may be achieved by shifting 1 h of time spent being sedentary to light physical activity, would reduce the risk of kidney dysfunction by 20%.

In conclusion, among a cohort of individuals with recently diagnosed diabetes, we observed that increases in sedentary time over 4 years were associated with increases in serum creatinine, while increases in total physical activity energy expenditure were associated with reductions in serum creatinine and a lower risk of CKD. Encouraging patients with Type 2 diabetes to increase their overall physical activity and decrease sedentary time may have beneficial effects on kidney function.

## Funding sources

The trial is supported by the Medical Research Council (grant reference no. G0001164), the Wellcome Trust (grant reference no. G061895), National Health Service R&D support funding (including the Primary Care Research and Diabetes Research Networks) and National Institute of Health Research under its Programme Grants for Applied Research scheme (RP‐PG‐0606‐1259). SJG was a member of the NIHR School for Primary Care Research. The Primary Care Unit is supported by NIHR Research funds. The views expressed in this publication are those of the authors and not necessarily those of the National Health Service, the NIHR, or the UK Department of Health. VYG was supported by a Global Scholarship Programme for Research Excellence‐CNOOC grant from the Chinese University of Hong Kong.

## Competing interests

None declared.

## Author contributions

Research idea and study design: VYG, SJG, RKS; data analysis and interpretation: VYG, SB, UE, SJG, RKS; statistical analysis: VYG; supervision or mentorship: SJG, RKS. Each author contributed important intellectual content during manuscript drafting or revision and accepts accountability for the overall work by ensuring that questions pertaining to the accuracy or integrity of any portion of the work are appropriately investigated and resolved. SJG takes responsibility that this study has been reported honestly, accurately, and transparently; that no important aspects of the study have been omitted; and that any discrepancies from the study as planned have been explained.

## Appendix

The ADDITION‐ Plus team included: R. Amin, G. Baker, R. Barling, M. Betts, A. Dickinson, J. B. Echouffo Tcheugui, U. Ekelund, F. Finucane, S. Mayle, J. Mitchell, P. Roberts, L. Sargeant, M. Sims, K. Westgate, F. Whittle and the Field Epidemiology, Data Management, IT, Physical Activity, Technical and Study Coordination teams (Medical Research Council [MRC] Epidemiology Unit, Cambridge, UK); J. Argles, R. Bale, R. Barling, S. Boase, J. Brimicombe, R. Butler, T. Fanshawe, P. Gash, J. Grant, S. Griffin, W. Hardeman, I. Hobbis, A. L. Kinmonth, T. McGonigle, N. Popplewell, A. T. Prevost, J. Smith, M. Smith, S. Sutton, F. Whittle and K. Williams (General Practice and Primary Care Research Unit, University of Cambridge, Cambridge, UK). The General Practice and Primary Care Research Unit at the University of Cambridge (GP) and the MRC Epidemiology Unit in Cambridge jointly coordinated the baseline and one year follow‐up phases of the study.
